# A challenging laboratory case due to inappropriate sampling through an antiseptic barrier cap

**DOI:** 10.11613/BM.2026.021001

**Published:** 2026-06-15

**Authors:** Giovanni Introcaso, Giorgia Piras, Giorgia Mandelli, Sabrina Frisoli, Fabio Bottari

**Affiliations:** 1 Clinical Laboratory Unit, Centro Cardiologico Monzino IRCCS, Milan, Italy; 2 Intensive Care Unit, Centro Cardiologico Monzino IRCCS, Milan, Italy

**Keywords:** alcohol contamination, analytical interferences, hematological analyzer, inappropriate sampling, preanalytical phase

## Abstract

Antiseptic barrier caps are currently used as novel tools in critically ill patients to achieve sterility of the blood sampling site and to prevent central line-associated bloodstream infections in the intensive care unit. A clinical and laboratory case of analytical interferences on erythrocytes, platelets and leukocytes due to the isopropyl alcohol contained in the antiseptic barrier cap is described. An aged man with history of heart valve disease was monitored for hematological parameters after surgery of mitral and tricuspid valve replacement. Leukocyte scattergrams changes on the Sysmex XN 2000 analyzer with the suspicion of platelet clumps were observed. After investigation on sampling procedure, antiseptic barrier cap of an arterial access (BD PureHub disinfecting cap) was used. Another sample has been requested, and the analysis of complete blood count and peripheral blood smear confirmed the presence of clumped microerythrocytes and erythrocytes with damaged membranes. In addition, differential leukocyte count revealed an overestimation of eosinophils by 10%. A new sample taken from venous access provided accurate results without alterations. The preanalytical error generated during blood sampling highlights the importance of correct use of biomedical devices. Furthermore, hematology analyzer technology represents a promising tool for assessing sample quality.

## Graphical abstract

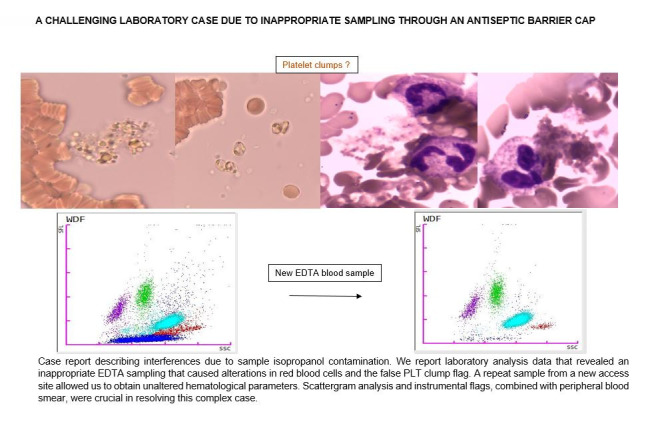

## Highlights

• Blood sampling is a process that can generate preanalytical errors

• Isopropanol contamination alters red blood morphology and cytometry

• Preanalytical error detected by hematology analyzers could be confirmed by peripheral blood smear

• Collaborative work between nurse and laboratory teams improves quality results

## Introduction

The present case report describes a laboratory challenge in identifying a preanalytical error and an example of effective collaboration between the clinical laboratory and the inpatient departments. Antiseptic barrier caps are currently used as novel tools in critically ill patients to achieve sterility of the blood sampling site then to prevent central line-associated bloodstream infections in the intensive care unit (ICU) (*1*). Recommendations and guidelines have been formulated by the Centers for Disease Control and Prevention (CDC) to prevent the common problem of hospital-acquired infections (*2*). Patients are treated in critical care settings such as ICUs, where bundled protocols are increasingly required, such as the septic bundle protocol. Furthermore, bedside care and treatment for critically ill patients requires special attention among healthcare workers, and standardization of therapeutic and care procedures is required.

Hematological analyzers provide a large amount of potentially useful data with the main parameters (*i.e.* complete blood count (CBC), leukocyte differential count, and hemoglobin (Hb) measurement). These parameters, called “research use only” (RUO), can provide useful information not only for pathologies but also about sample quality (*3*-*6*).

In this clinical case we demonstrated analytical interferences on the analyses of erythrocytes, platelets and leukocytes due to the isopropyl alcohol contained in the antiseptic cap of a luer connector. Instrumental flags along with analysis of scattergrams allowed detection of interferences in the ethylenediaminetetraacetic acid (EDTA) blood sample. An aged man with a history of heart valve disease was hospitalized for mitral and tricuspid valve replacement surgery. However, the patient underwent coronary artery bypass grafting under normothermic extracorporeal circulation and cardioplegic arrest. Two units of packed red blood cells were transfused. Biochemical and hematological tests were requested daily showing postsurgical anemia and high inflammatory markers. On the fourth day of ICU monitoring, an EDTA sample was processed in the laboratory for CBC. This common laboratory test allows monitoring the immunological response, the state of anemia and hemostasis in the post-operative period.

In addition, modern analyzers can be useful to define preanalytical conditions of anticoagulated blood samples that could influence diagnostic test results. Our clinical case highlights the role of new analytical technologies and laboratory procedure in improving the quality of diagnosis or clinical monitoring in hematology.

## Laboratory analyses

**Figure 1:**
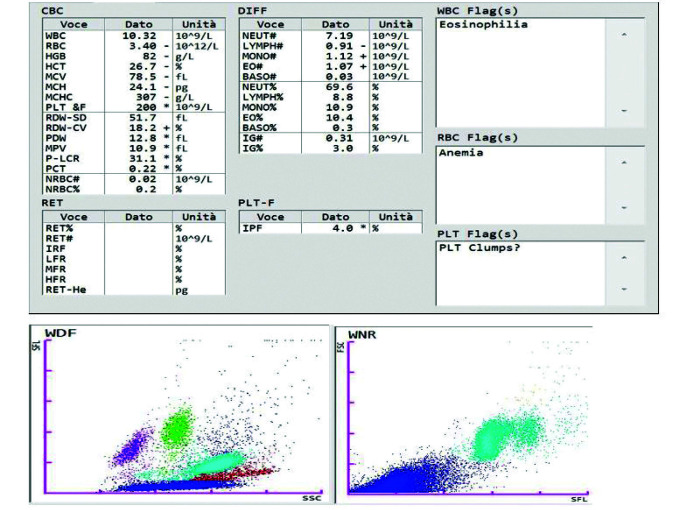
Complete blood count results of the isopropanol contaminated sample. The scattergrams show interferences in both the White cell differential (WDF) and White cell nucleated red (WNR) channels (blue color). Eosinophils were not well separated (red color) determining an overestimation. Scattergram dispersion generated the false platelet clumps (PLT Clumps?) flag.

**Figure 2:**
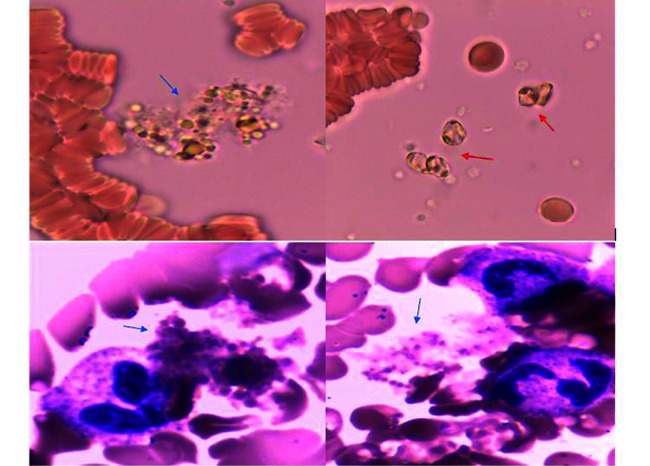
Red blood cells with alterations and membrane damage (red arrows). Microerythrocytes agglutination resulted as effect of isopropanol 70% (blue arrows). Above, the fresh blood smear. Below, the May-Grünwald smear (1000X).

**Figure 3:**
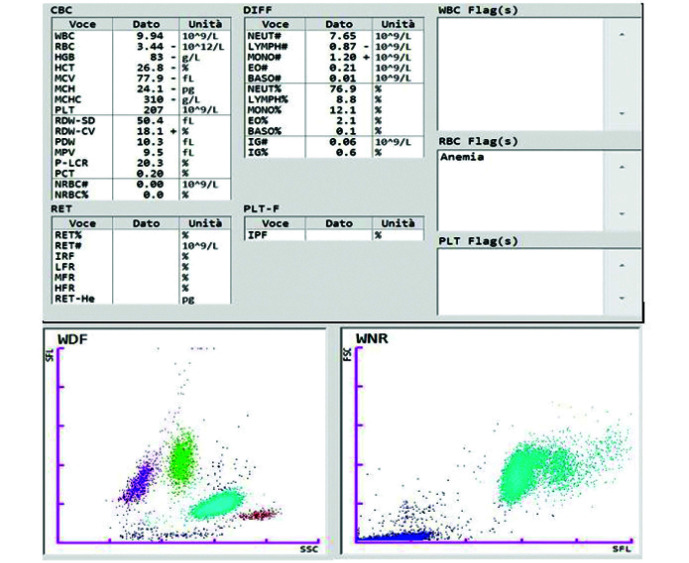
Complete blood count results of the second sample. Leukocyte populations are well separated without interferences and instrumental flags.

During routine work, the technician and the laboratory specialist observed changes in leukocyte scattergrams of the patient sample on the Sysmex XN 2000 analyzer (Sysmex Corporation, Kobe, Japan) (Figure 1). Afterwards, a peripheral blood smear (PBS) on fresh whole blood and May-Grünwald Giemsa staining was scheduled for the suspicion of platelet (PLT) clumps highlighted by the PLT flag: PLT clumps? on the analyzer screen. The EDTA sample was macroscopically inspected and showed no abnormalities; a repeated PLT count on the fluorescent channel (PLT-F) confirmed the previous PLT count without instrumental flags. No platelet aggregation was detected with this channel. However, analysis of PBS excluded the presence of PLT agglutinates or aggregates. A careful evaluation of the smear revealed damaged erythrocytes and microerythrocytes that agglutinated and simulated on the analyzer agglutinated platelets (Figure 2). The damaged red blood cells produced hemolysis which however did not cause a significant change in the patient’s hemoglobin concentration (Figure 1 and Figure 3). Fresh whole blood smear allowed us to quickly highlight anomalous morphological features and to recognize the alterations of the red blood cells. A blood smear with May-Grünwald Giemsa technique confirmed the previous criticisms (Figure 2). Alterations of leukocyte scattergrams generated the PLT clump flag on the white cell differential (WDF) and white cell nucleated red (WNR) channels and the suspicion of leukocyte misclassification. For this reason, we prompted the control of leukocyte differential count by manual microscope method revealing an analytical overestimation of eosinophils of 10%. A complete blood count on a new sample from the ICU showed these data without quantitative and qualitative alterations (Figure 3).

## What happened?

During daily post-surgery patient monitoring in ICU, blood samples for hematological tests were obtained from a luer connector. In the laboratory, scattergram analysis of differential leukocyte and instrumental flags suggested the presence of PLT agglutinates or the possible infection due to fungi or microorganisms. However, a PBS as additional investigation was performed. We called the ICU asking for clinical information and if there were any problems with the sampling. A variation of the sampling procedure has been reported: the cap has been used directly without the backflow valve (BD PureHub disinfecting cap, Becton, Dickinson and Company, Franklin Lakes, USA). This procedure may have generated sample contamination. The disinfectant may therefore have been accidentally aspirated into the EDTA tube during collection and a small amount of 70% isopropanol could have therefore contaminated the sample, causing cytological damage of red blood cells. At this point, to verify the contamination hypothesis, a new blood sample taken from another access route was then requested. Results on a new EDTA sample with normal outcomes (Figure 3) confirmed the preanalytical hypothesis. The information obtained by a phone call with the ICU nurse asking for explanations on the sampling procedures has been very important. It allowed us to suppose a variation of the sampling procedure used in ICU clarifying that the observed analytical anomalies could be due to the sampling phase. The explanation of the use of the luer connector and the cap with 70% isopropanol disinfectant allowed us to resolve this challenging case due to a preanalytical error of inappropriate sampling. A careful team evaluation of the analytical and preanalytical data allowed us to correct the sampling error before the clinical results validation and reporting.

## Discussion

Technology of modern hematological analyzers using laser beam on a biological sample by light scattering allowed us to obtain important clinical information along with PBS evaluation (*6*, *7*). These analyzers automatically provide new data defined cell population data (CPD) or leukocyte CPD (*5*-*8*). However, recent studies aimed to determine the accuracy of instrumental flags on Sysmex analyzers for detecting platelet clumps (*9*-*11*). Therefore, preanalytical evaluation of the blood sample from which the quality of the results derives is increasingly investigated. Flow cytometry used in hematology analyzers could provide automated analysis of sample suitability prior to analytical parameter analysis. Our work underlines the possibility of preanalytical error detection using instrumental flags with biological interferences; then, the instrument highlights the possible issue that requires further investigation. Indeed, in this case report, we described the detection of errors during a blood sampling through carefully evaluated platelet flags and leukocyte scattergrams. To date, microscopic analysis using a PBS evaluation was essential to clarify the analyzer flags: a suspicion of morphological alteration or sample artefacts requires a PBS confirmation. In fact, red blood cell damage and related parameter alterations due to sample contamination by isopropyl alcohol were highlighted. In literature there are numerous cases of contamination of samples for microbiological and genetic tests. Our report first reported a case of preanalytical error due to contamination through an antiseptic barrier, although many cases of red blood cell alterations are known. For instance, the effects of cephalosporins on red blood cells are well known. Ceftriaxone, a widely used antibiotic, is one of the most common drugs to cause drug-induced immune hemolytic anemia with alterations of RBC derived-parameters (*11*, *12*). Today, antiseptic barrier caps are currently used as biomedical devices to optimize blood sampling while reducing bacterial infections. Here, we have shown an occasionally improper use of these devices determining an inappropriate sampling with potential incorrect results and patient misclassification. In our laboratory, there is currently a procedure for preanalytical evaluation but it is operator dependent. As a corrective action, standardization of sampling procedures in the department and a careful validation of the preanalytical phase by the analyzer might be implemented. Flow cytometry seems to be a promising technique for identifying inappropriate samples.

## What YOU should do in your laboratory to prevent such errors

The BD PureHub disinfectant cap is currently used as novel biomedical device, designed to provide high-level protection for needle-free luer connectors. These devices are cornerstones for care reducing the risk of bacterial contamination, both as a rapid disinfectant and as a protective barrier between catheter access points. Although rare events, inappropriate use of these biomedical devices can occur in clinical practice, especially in critical conditions. For this reason, shared procedures between operators and protocols aimed at standardizing healthcare are essential. Collaboration between laboratory and ICU was crucial in detecting non-conformity. For this, continuous education for both laboratory technicians and ICU staff can help prevent errors. Besides, specific protocols for preanalytical evaluation in clinical laboratories are required. In this sense, the definition of instrumental flags to detect preanalytical errors will be increasingly important. Fluorescent platelet channel on Sysmex analyzers detecting PLT aggregation or PLT agglutination is promising. Careful sample evaluation remains crucial to increase the quality of clinical data. Standardization of preanalytical phase through new technological insights is required to improve the total quality. Furthermore, implementation of validation rules on hematology analyzers may improve the detection of preanalytical errors helping laboratory teamwork.

## Data Availability

The data generated and analyzed in the presented study are available from the corresponding author on request.
